# Stroke Severity in Transcatheter Aortic Valve Implantation Versus Surgical Aortic Valve Replacement: A Systematic Review and Meta-Analysis

**DOI:** 10.1016/j.jstrokecerebrovasdis.2021.105927

**Published:** 2021-09

**Authors:** Pádraig Synnott, Robert P Murphy, Conor Judge, Maria Costello, Catriona Reddin, Karen Dennehy, Elaine Loughlin, Andrew Smyth, Darren Mylotte, Martin J. O'Donnell, Michelle Canavan

**Affiliations:** a HRB-Clinical Research Facility, NUI Galway, Galway, Ireland; b Translational Medical Device Lab, NUI Galway, Galway, Ireland; c Wellcome Trust – HRB, Irish Clinical Academic Training, Ireland

**Keywords:** TAVI, SAVR, Stroke, Stroke severity, Meta-analysis

## Abstract

**Objectives:**

An assessment of the comparative incidence of fatal or disabling stroke may influence choice of intervention for patients with severe aortic stenosis. We explored whether transcatheter aortic valve implantation (TAVI) is associated with a lower incidence of fatal or disabling stroke, compared to surgical aortic valve replacement (SAVR).

**Materials & Methods:**

We classified stroke into two categories; fatal or disabling, or non-disabling, and completed meta-analyses for each. We explored randomised controlled trials to assess the effect publication year, predicted operative risk, and route of TAVI access.

**Results:**

There was no difference between treatment groups per 100 person years of follow up for disabling or non-disabling stroke outcomes. In a stratified analysis by year of publication, there was a lower rate of fatal or disabling stroke with TAVI in trials published after 2015, compared to those published in 2015 or before (p-interaction = 0.01 at 30 days). Higher proportions of transfemoral route access (>90%), more common in recent trials, were associated with a lower rate of fatal or disabling stroke (p-interaction = 0.03 at 30 days). Lower average surgical risk scores were associated with lower rates of fatal or disabling stroke (p = 0.02 at 30 days).

**Conclusion:**

We found that treatment of aortic stenosis with TAVI compared with SAVR was not associated with an overall reduced risk in fatal or disabling stroke. Subgroup analyses suggested a lower risk of fatal or disabling stroke with TAVI in situations which reflect contemporary practice.

## Introduction

In patients with severe aortic stenosis, who are at low or intermediate operative risk, transcatheter aortic valve implantation (TAVI) or surgical aortic valve replacement (SAVR) may be performed. Previous meta-analyses have found that compared with SAVR, TAVI may have similar or reduced early and midterm all-cause mortality outcomes in patients across the range of low to high surgical risk, with a transfemoral approach providing more favourable outcomes.^1^^,^^2^

Choice of intervention in intermediate and lower risk patients is dependent on numerous factors, including availability of transfemoral access, anatomical suitability and age, as well as an assessment of the comparative incidence of stroke. One of the most feared complications of TAVI is stroke because of its associated severe disability and high mortality.^3^ A meta-analysis of mainly observational studies found that stroke was the third leading individual cause of death within 30 days in those using the Edwards SAPIEN Valve.^4^ Previous meta-analyses^3^^,^^5^^,^^6^ have focused on comparing risk of absolute stroke event rates between both groups, but have not addressed the effect on stroke severity.

The publication of two randomised controlled trials in 2019^7^^,^^8^ and the availability of extended follow-up of studies^8^, ^9^, ^10^, ^11^ and final reports on neurological outcomes^12^ prompted this updated meta-analysis comparing stroke specific outcomes, with a focus on short, intermediate, and long term functional stroke outcomes of adult patients with severe aortic stenosis, undergoing either TAVI or SAVR, in randomised controlled trials. We compared total stroke rates, with a focus on stroke severity (ie fatal or disabling stroke), as our primary outcomes. Exploring a potential differential effect of TAVI (versus SAVR) in relation to stroke severity is of clinical importance to patients and clinicians. The criteria for TAVI has expanded to incorporate lower surgical-risk patients^13^ and knowledge about what treatment strategy offers the lowest risk of disabling stroke may influence individual decision making.

## Methods

We performed a systematic review and meta-analysis which adhered to the Preferred Reporting for Systematic Reviews and Meta-analyses (PRISMA) guidelines.^14^ A PRISMA flow diagram was completed to outline the screening process for eligible trials (eFig. 1). The protocol was registered with PROSPERO (Registration Number 42020153165). We included all randomised controlled trials (RCTs) which have investigated TAVI versus SAVR in severe aortic stenosis, including comparisons in low, intermediate and high-risk surgical candidates.

**Fig. 1:**
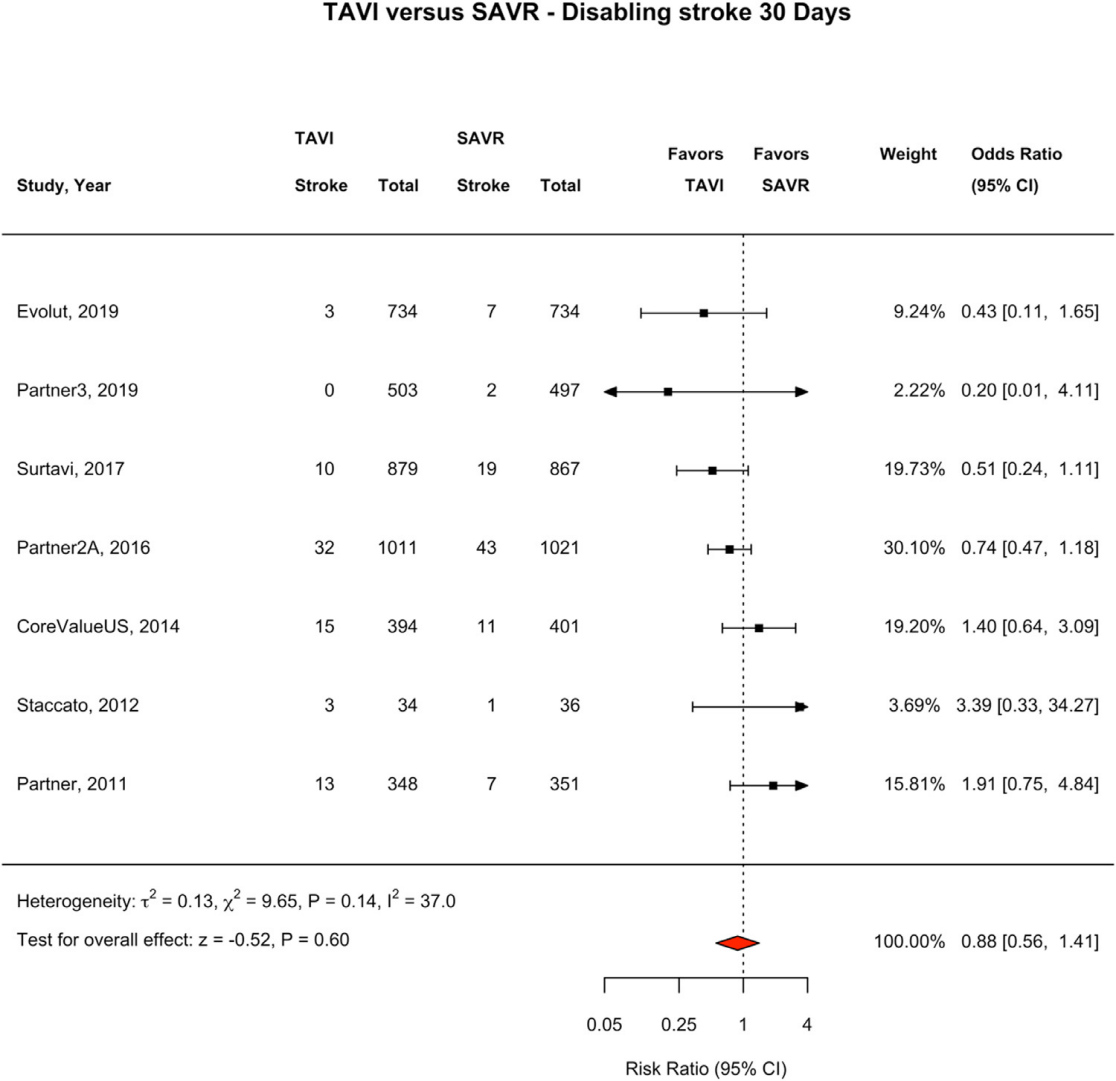
30 day Fatal or Disabling Stroke: TAVI vs SAVR. Fig. 1. Forest Plot showing fatal or disabling stroke events by treatment group at 30 days. The square bars represent the mean values and 95% confidence interval of the effect sizes, while the size of the squares reflects the weight of the studies. The combined effect appears as a diamond and the vertical dashed line represents the line of no effect. TAVI - transcatheter aortic valve implantation, SAVR – surgical aortic valve repair, CI – Confidence Interval.

### Data Sources and Searches

We extracted data from two previous meta-analyses: one of randomised controlled trials comparing clinical outcomes in adults with severe aortic stenosis undergoing either TAVI or SAVR,^1^ and the other of outcomes in specifically high surgical risk patients undergoing either TAVI or SAVR.^2^ We considered these meta-analyses of sufficiently high quality to avoid the need to repeat them and to reduce research waste we restricted our search dates from August 1^st^ 2016 onwards.^15^ We completed a search of Medline, Medline in-process, EMBASE and Cochrane Central from August 1^st^ 2016 to October 31^st^ 2019 using a combination of keywords and MeSH terms for ‘aortic stenosis” AND ‘valve replacement’ using the sensitive search filters for therapeutic interventions developed by the Health Information Research Unit at McMaster University.^16^ There were no language or publication type restrictions. We also completed searches of all references from all included studies.

### Outcomes

Our primary outcome was a comparison of fatal or disabling strokes (Modified Rankin Score of 3–6), and non-disabling strokes (Modified Rankin Score 1,2). We reported outcomes at 30 days, at one year and longest follow up. We reported longest follow up per trial as 100 person years of follow up. Secondary outcomes included all stroke outcomes and subgroup analysis of TAVI access route, trial surgical risk category, duration of antiplatelet therapy and a comparison between earlier trials (published in 2015 or before) and more recent trials (after 2015). Trial surgical risk category was determined using the mean reported score from The Society of Thoracic Surgeons Predicted Risk of Mortality (STS-PROM) to predict risk of death at 30 days post-procedure.^17^

### Data Extraction

Primary results were extracted from the original RCTs, but follow up studies were reviewed and data extracted from these on longer term outcomes studies^8^, ^9^, ^10^, ^11^ and final reports with further details on neurological outcomes.^12^ Data were extracted separately for disabling stroke, non-disabling stroke, and total stroke. If there was a discrepancy between the addition of non-disabling stroke and disabling stroke with total stroke, we extracted what the primary paper reported for total stroke.^7^^,^^18^^,^^19^ Data was extracted as absolute numbers for all trials except one, where data was calculated from Bayesian estimated incidences.^7^ We (PS, RM) completed primary data extraction independently for each paper to confirm accuracy and resolved any inconsistencies by third party consensus (CJ).

### Data synthesis and analysis

For our primary outcome at 30 days and 1 year we calculated odds ratios (OR) and 95% confidence intervals (CI) from individual studies. We also reported outcome per 100 person years of follow up. Weighted pooled treatment effects were calculated using a random effects model. Where possible, we meta-analysed the intention to treat population from trials. Statistical analysis was performed using the Metafor package on R Statistical Software.^20^ We explored effect-modification for the following variables: TAVI access route, year of study publication, mean age of study participants, and categorization of risk using the STS-PROM risk score. Comparisons were 2-tailed using a P ≤ 0.05 threshold for all analyses apart from subgroup interactions where we used a P ≤ 0.1 threshold.^21^

### Risk of Bias Assessment

To assess the study quality we assessed risk of bias using the Cochrane Risk of Bias 2 tool.^22^ Each study was judged as being either at low risk of bias, some concerns or high risk of bias. Two reviewers (PS and RM) assessed bias and reviewers resolved conflicts through consensus with a third party (CJ).

## Results

We identified 8 trials.^7^^,^^8^^,^^18^^,^^19^^,^^23^, ^24^, ^25^, ^26^ with a total of 8,090 participants which reported stroke incidence, or incidence of fatal, disabling and non-disabling strokes on follow-up. Of these, 4,048 participants were randomised to TAVI, and 4,042 participants randomised to SAVR. Baseline characteristics of participants involved in each trial is summarised in Table 1. The mean age of trial participants was 79.5 years. Four studies were in low-risk populations,^6^^,^^7^^,^^19^^,^^20^ two in intermediate risk populations,^19^^,^^24^ and two in high risk populations.^18^^,^^26^ The median duration of follow-up was 3.5 years, the longest follow-up was 5 years,^18^^,^^19^^,^^23^^,^^26^ and the shortest follow-up was 3 months.^25^

**Table 1:** Study Characteristics

Study	Type of Valve Used	Surgical Risk Stratification	Gender Women %	Mean Age	TAVI Randomised (n)	SAVR Randomised (n)	Transfemoral Approach %	TAVI prior CVD %	SAVR prior CVD %	Coronary Intervention (PCI)	Coronary Intervention (CABG)	Antiplatelet Anticoagulant Regimen
Partner1A (2011)	Sapien heart-valve system	High	42.7	84.1	348	351	70.1	29.3	27.4	NR	NR	DAPT 6/12
Staccato (2012)	Sapien heart valve system	Low	70.0	81.0	34	36	0.0	2.9	2.8	0	1	DAPT (duration unspecified)
CoreValueUS (2014)	CoreValve prosthesis	High	46.7	83.4	394	401	81.2	25.6	26.0	1	17	DAPT 3/12 OR aspirin + VKA
Notion (2015)	CoreValve prosthesis	Low	46.7	79.1	145	135	94.5	16.6	16.3	0	1	DAPT 3/12 OR clopidogrel +VKA 3/12 then lifelong aspirin
Partner2A (2016)	Sapien XT valve system	Intermediate	45.4	81.6	1011	1021	76.7	32.1	31.0	39	137	DAPT 1/12, OR Aspirin plus VKA/DOAC
Surtavi (2017)	CoreValve prosthesis/EvolutR prosthesis	Intermediate	43.2	79.8	879	867	91.9	13.4	13.5	125	176	DAPT 3/12 or antiplatelet plus warfarin
Evolut (2019)	CoreValve/EvolutR/EvolutPRO	Low	34.9	74.0	734	734	99.0	10.1	11.4	50	92	DAPT 1/12 then aspirin monotherapy
Partner3 (2019)	Sapien 3	Low	30.7	73.0	503	497	100.0	3.4	5.1	32	58	DAPT 1/12 OR aspirin and VKA/DOAC

Abbreviations: TAVI, transcatheter aortic valve implantation; SAVR, surgical aortic valve repair; PCI, percutaneous coronary intervention; CABG, coronary artery bypass graft; DAPT, dual antiplatelet therapy; VKA, vitamin K antagonist; DOAC, direct oral anticoagulant; CVD, cerebrovascular disease

### Stroke Outcomes at Longest Follow-Up

In addition to 30 day and 1 year outcomes, longest follow-up with a breakdown of stroke subtypes was reported at two years for two trials,^10^^,^^28^ and at five years for two trials.^9^^,^^29^ There was no difference in the incidence of fatal or disabling strokes per 100 person years of follow up between TAVI and SAVR (0.89 vs 1.52, incidence difference 0.02 (-.32 – 0.35). There was no difference in the incidence of non-disabling strokes per 100 person years of follow up between TAVI and SAVR (1.35 vs 1.30, incidence difference .07 (-.23 – 0.36). An analysis of all stroke outcomes reported between 1 year and year 2 of follow up showed no statistically significant difference in strokes in the TAVI and SAVR population (OR 1.09, CI 0.57–2.10, I^2^ = 36) (eFig. 2).

**Fig. 2:**
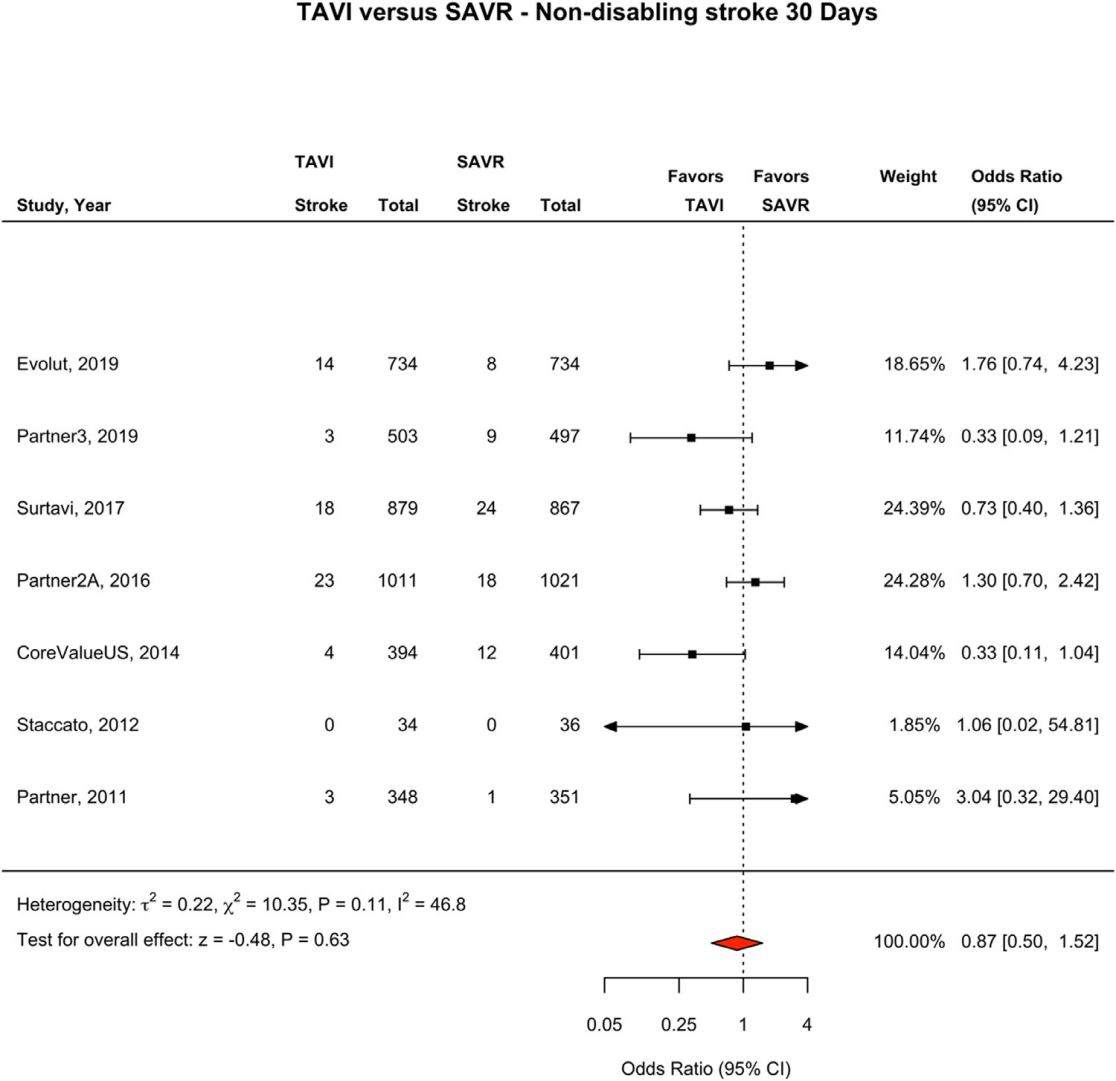
30 day Non-Disabling Stroke: TAVI vs SAVR. Fig. 2, 30-day non-disabling stroke. Forest Plot showing non-disabling stroke events by treatment group at 30 days. The square bars represent the mean values and 95% confidence interval of the effect sizes, while the size of the squares reflects the weight of the studies. The combined effect appears as a diamond and the vertical dashed line represents the line of no effect. Abbreviations: TAVI, transcatheter aortic valve implantation; SAVR, surgical aortic valve repair; CI, Confidence Interval.

### Stroke Outcomes at 30 days

At 30 days, seven of the eight RCTs (n=7) reported a breakdown of fatal or disabling stroke and non-disabling stroke.^7^^,^^8^^,^^18^^,^^19^^,^^24^, ^25^, ^26^ At 30 day follow up, 166 fatal or disabling strokes had been reported, 76 (1.95%) in the TAVI population and 90 (2.30%) in the SAVR population, accounting for 55.5% of all strokes in the first 30 days. 137 non-disabling strokes had been reported, 65 (1.67%) in the TAVI population and 72 (1.84%) in the SAVR population, accounting for 45.8% of all strokes in these seven trials. At 30 days, 305 total stroke events had been reported, 141 (3.48%) in the TAVI population, and 164 (4.06%) in the SAVR population. There was no significant difference between groups in the incidence of fatal or disabling stroke (OR 0.88, CI 0.56–1.41, I^2^ = 37.0), non-disabling stroke (OR 0.87, CI 0.50–1.52), or all stroke Figs. 1 and 2, eFig. 3).

**Fig. 3:**
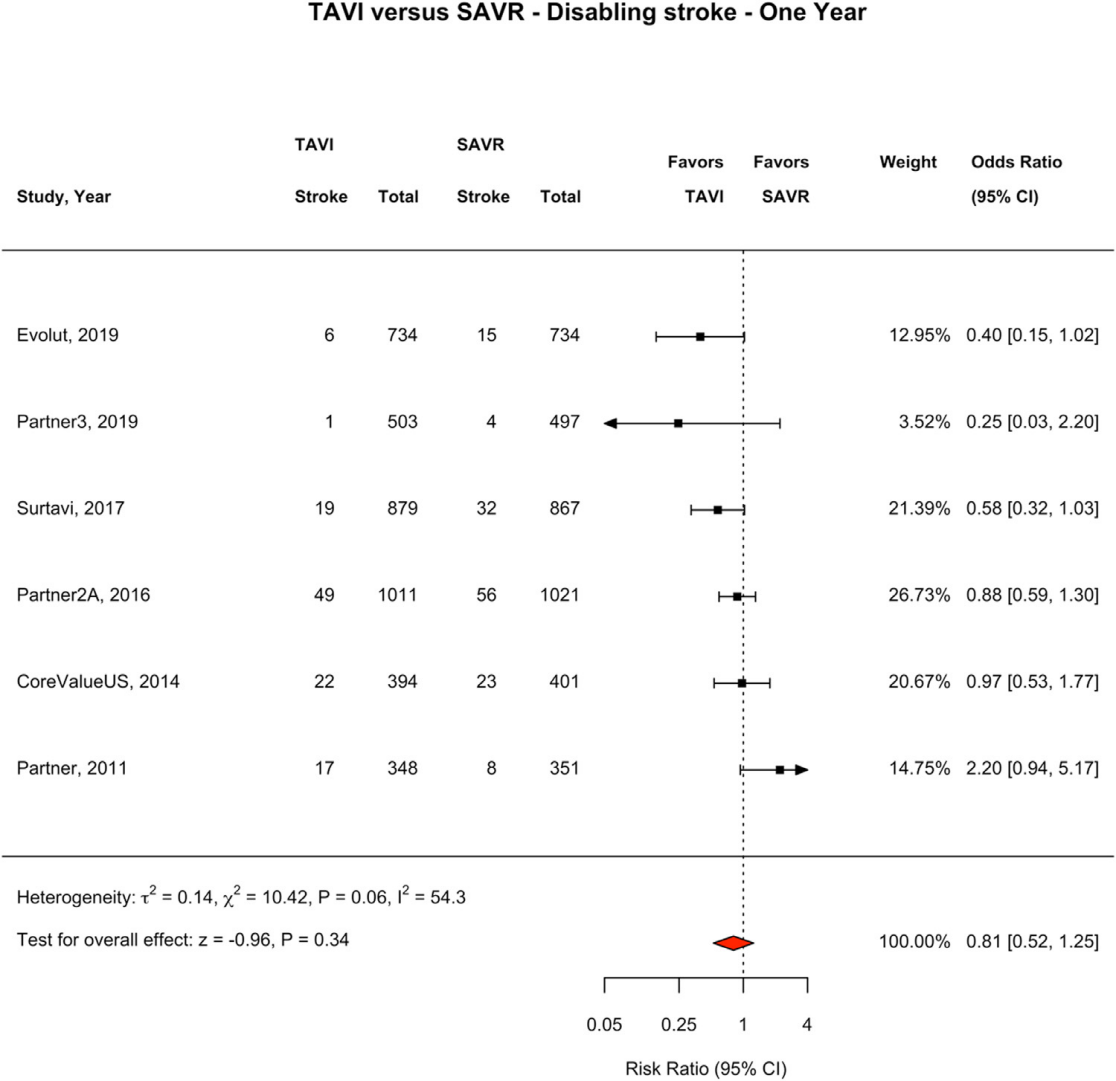
1 year Disabling Stroke: TAVI vs SAVR. Fig. 3. Forest Plot showing fatal or disabling stroke events by treatment group at one year. The square bars represent the mean values and 95% confidence interval of the effect sizes, while the size of the squares reflects the weight of the studies. The combined effect appears as a diamond and the vertical dashed line represents the line of no effect. TAVI - transcatheter aortic valve implantation, SAVR – surgical aortic valve repair, Int- Intervention, CI – Confidence Interval.

### Stroke Outcomes at One Year

**Fig. 4:**
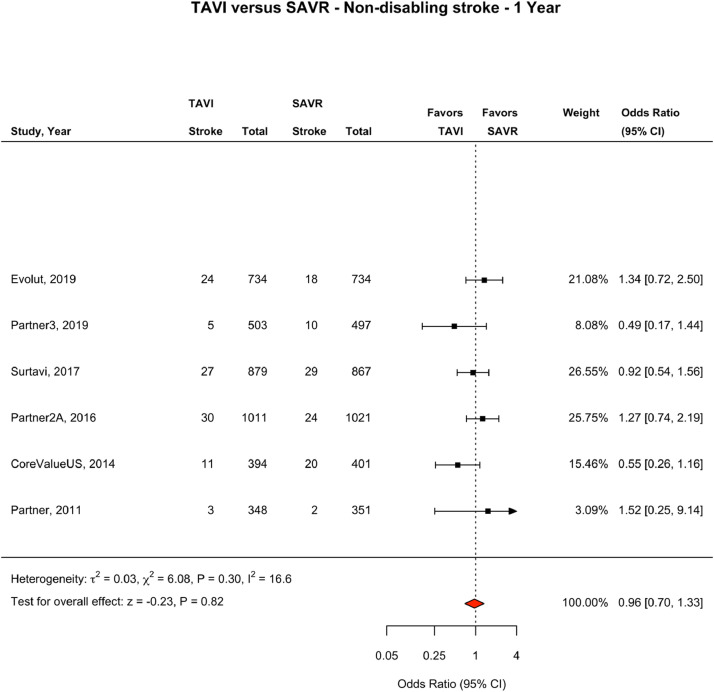
1 year Non-Disabling Stroke: TAVI vs SAVR. Fig. 4. One-year non-disabling stroke events. Forest Plot showing non-disabling stroke events by treatment group at one year. The square bars represent the mean values and 95% confidence interval of the effect sizes, while the size of the squares reflects the weight of the studies. The combined effect appears as a diamond and the vertical dashed line represents the line of no effect. Abbreviations: TAVI, transcatheter aortic valve implantation; SAVR, surgical aortic valve repair; CI, Confidence Interval

At one year follow up, six of the eight RCTs (n=6) reported a breakdown of fatal or disabling stroke and non-disabling stroke.^7^^,^^8^^,^^18^^,^^19^^,^^24^^,^^26^ At one year follow up, 245 fatal or disabling strokes had been reported, 113 (2.89%) in the TAVI population and 132 (3.38%) in the SAVR population, accounting for 54.2% of all strokes reported in these six trials. 203 non-disabling strokes were reported, 100 (2.56%) in the TAVI population and 103 (2.64%) in the SAVR population, accounting for 45.2% of all strokes reported in these six trials. At one year, 452 total strokes had been reported, 215 (5.31%) in the TAVI population and 237(6.07%) in the SAVR population, (OR 0.90, CI 0.74-1.09, I^2^ = 0.0). There was no significant difference between groups in the incidence of fatal or disabling stroke (OR 0.85, CI 0.58–1.23, I^2^ = 39.4), non-disabling stroke (OR 0.96, CI 0.70–1.33, I^2^ = 16.6), or all stroke (Figs. 3 and 4, eFig. 4).

### Subgroup Analyses

**Fig. 5:**
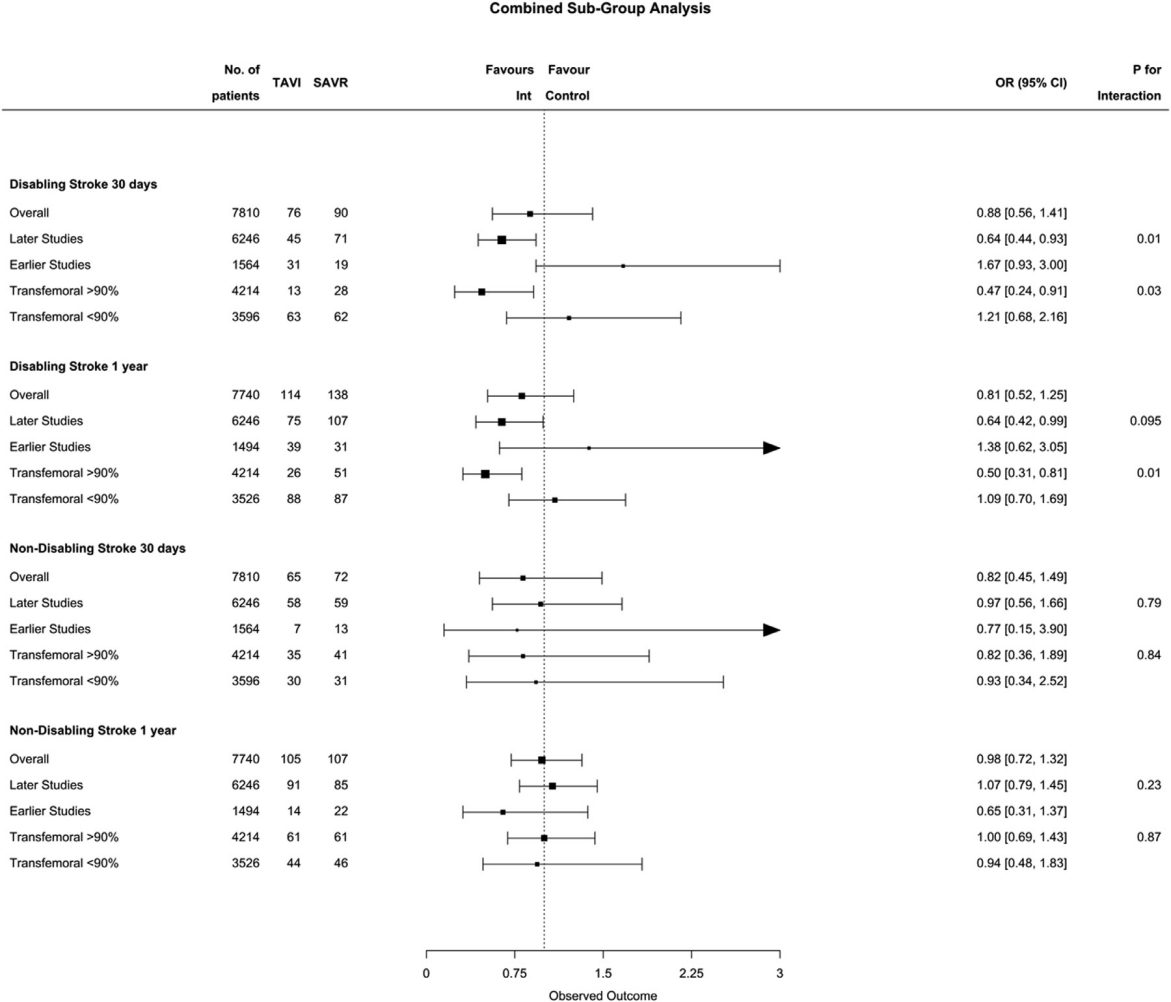
Combined Forest Plots for Subgroup Analysis. Fig. 5. Combined Forest Plot showing the subgroup analysis by timing of studies (earlier vs later) and proportion transfemoral access (<90% of total patients in study with transfemoral access vs >90% of total patients in study with transfemoral access). The combined effect appears as a square bar, with 95% confidence intervals of the effect sizes, which the area of the squares reflects the weight of the studies. The vertical dashed line represents the line of no effect. TAVI - transcatheter aortic valve implantation, SAVR – surgical aortic valve repair, Int- Intervention, OR – Odds Ratio, CI – Confidence Interval.

Results of subgroup analyses are presented in Fig. 5. We undertook a sensitivity analysis exploring if there was a differential treatment effect depending on the year the studies were conducted, comparing the earlier four RCTs with the more recent four RCTs. A differential effect was observed for both disabling stroke at 30 days and one year.

Among trials conducted from 2016 onwards, the risk of fatal or disabling stroke was lower with TAVI at 30 days (OR 0.64, CI 0.44–0.93) and one year (OR 0.64, CI 0.42 0.99), with no difference in those conducted before 2016 at 30 days (OR 1.67, CI 0.93-3.0) or one year (OR 1.38, CI 0.62–3.05). The p for interaction was significant at 30 days (*P*=0.01) and at one year (p= 0.095) for disabling stroke, with lower risk of stroke in the studies which were done more recently.

In trials with a high proportion of transfemoral access (>90%), the risk of fatal or disabling stroke was lower with TAVI at 30 days (OR 0.47, CI 0.24–0.91) and at one year (OR 0.50, CI 0.31–0.81), with no difference compared to trials using lower proportions of transfemoral access at 30 days (OR 1.21, CI 0.68–2.16) or one year (OR 1.07, CI 0.7–1.69). There was evidence of a statistically significant interaction (P=0.03 at 30 days and P=0.01 at 1 year). A high proportion of transfemoral access was favoured in the more contemporary trials (Table 1).

**Fig. 6:**
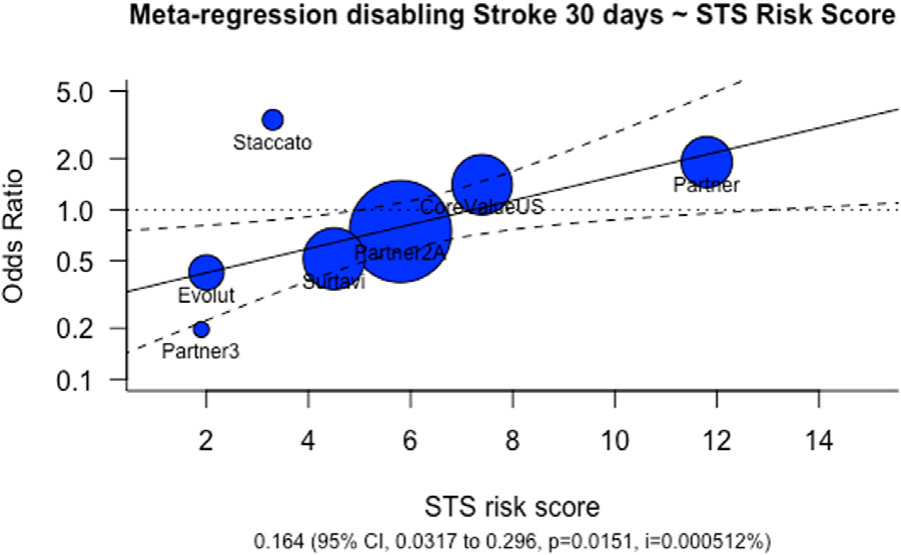
Meta-Regression Analysis Disabling Stroke 30 days ~ STS Risk Score. The solid line represents the regression line for the analysis. Individual studies are represented by circles, with the size of the circle being inversely proportional to the variance of the estimated treatment effect. STS Risk score represents the mean STS risk score of participants in each study. Odds ratio represents risk of disabling stroke at 30 days. Abbreviations: CI, Confidence Interval; STS, Society of Thoracic Surgeons.

There was significant negative correlation between mean age of trial participants and year of study publication (Pearson correlation coefficient = -0.85). Meta-regression analysis suggested that there was an association with lower average STS-PROM risk scores and less fatal or disabling strokes at 30 days (Fig. 6, p = 0.01).

### Risk of Bias

Risk of bias was assessed using the Cochrane Risk of Bias 2 Tool (eFigures 5 & 6), graded as “high risk”, “low risk” or “some concerns”. The overall risk of bias was deemed high risk for the PARTNER1A Trial,^26^ low risk for the STACCATO trial,^25^ with some concerns identified in different domains for the remaining trials.^7^^,^^8^^,^^18^^,^^19^^,^^23^^,^^24^ The randomisation process was at low risk of bias for all trials. There were some concerns for risk of bias of outcome assessment for the NOTION trial^23^ and Partner 3 trial^8^ due to assessors being unblinded. NOTION was the only study to not include a breakdown of disabling versus non-disabling strokes so this lack of blinding of outcome assessors did not affect our primary outcome analysis. A sensitivity analysis without the Partner 3 trial did not change the results of our primary analysis. There were some concerns of bias due to missing outcome data in the Partner 2A trial^19^ as less than 90% of SAVR patients had 30 day follow-up, and low risk of bias for missing outcome data for all other trials. There were some concerns regarding risk of bias for deviations from intended interventions for 6 of 8 trials,^7^^,^^8^^,^^18^^,^^19^^,^^23^^,^^24^ and high risk of bias for the Partner 1A trial.^26^ This was caused by fewer patients in SAVR groups receiving the assigned intervention compared with TAVI groups. A sensitivity analysis was conducted excluding Partner 1A trial, which was deemed high risk of bias, and this did not affect overall stroke outcomes.

## Discussion

We performed a systematic review and meta-analysis of all RCTs comparing TAVI with SAVR for severe aortic stenosis, to determine whether stroke severity differed between interventions. Although we did not find an overall difference in fatal or disabling stroke risk among all trials, the incidence of fatal or disabling stroke appeared lower with TAVI, when analyses were confined to more recent trials, and/or those with the highest proportion undergoing a transfemoral TAVI approach.

Our meta-analysis completed an extended analysis of stroke risk and found evidence of fewer disabling strokes in TAVI (compared to SAVR) in recent randomised controlled trials. While recent meta-analysis have demonstrated a trend towards a lower rate of disabling stroke^6^ our results advance these findings by highlighting the clinical scenario where the optimum reduction in disabling stroke can be achieved. Our analysis suggests a differential effect on incidence of severe stroke, in favour of TAVI, in recent trials, which are also those with highest proportion of transfemoral approaches. Given that more recent trials are expected to better reflect contemporary clinical practice, our findings are likely to be clinically relevant, despite being derived from subgroup analyses. These observations might also lend some support to the contention that increased use of a transfemoral approach may be associated with less disabling stroke, although trials with increased rates were also those completed most recently, meaning that other differences may have accounted for our findings. Factors that may have accounted for improved safety of TAVI could include improved patient selection, operator experience and improved valve technology systems.^30^ A sensitivity analysis comparing trials using balloon expandable valves^8^^,^^19^^,^^25^^,^^27^ with self-expanding valves^7^^,^^18^^,^^23^^,^^24^ found no difference in stroke outcomes.

While the low number of trials in our analysis precluded a definitive exploration, we explored this heterogeneity through examining for the correlation between year of trial publication and mean age of trial participants. They were highly negatively correlated indicating that mean age of trial participants decreased as the more recent TAVI trials were published. This correlation reflects that more recent trials had younger patients, rather than reflecting there being a differential risk of more disabling strokes in older patients.

Our meta-analysis provides information that may be useful to clinicians and patients, when discussing management options for aortic stenosis. Our results could help inform shared decision making with patients at dedicated multidisciplinary ‘Heart Team’ discussions, as is recommended in latest guidelines.^31^ Stroke is among the most feared complications of patients undergoing TAVI, often more so than death,^32^ and optimal decision-making requires high-quality information on the competing risks and benefits of TAVI or SAVR.

Comparative rates of disabling and non-disabling stroke, and incidence of stroke at longest follow up have not been reported prior to this meta-analysis.^33^ We reported no difference in stroke incidences per 100 person years of follow-up across all stroke and in addition our subgroup analysis of stroke rates from year 1 to year 2 post procedure highlighted no differential treatment effect across procedure type in this time frame. While further data is required to confirm long-term durability of TAVI beyond five years^34^ our results give the most up to date analysis of comparative stroke risks. This is of particular importance because indications for TAVI have expanded, and TAVI is now endorsed as a standard therapy for lower risk patients with symptomatic aortic stenosis.^13^ Likewise the indications for TAVI may expand further, with trials ongoing assessing TAVI in low risk and younger patients, as well as the role of TAVI in asymptomatic severe aortic stenosis.^35^^,^^36^

Limitations of our meta-analysis include our inability to report stroke severity by primary stroke aetiology (ischaemic versus haemorrhagic), and report on the net clinical benefit related to stroke severity, for the composite of ischaemic stroke and ICH. Furthermore, we were unable to determine whether there was a differential effect between treatments by ischemic stroke aetiological subtypes or by topographical subtype (e.g. the Oxfordshire (Bamford) classification)^12^ which may have been of relevance in comparing stroke severity and disability. We did not have information on management of ischaemic strokes in trials, such as proportion who underwent thrombolysis or thrombectomy, which would have impacted on our outcomes of stroke disability. This may differ between groups, as recent SAVR is considered a contraindication to thrombolysis, whereas TAVI is a relative contraindication. As outlined, prolonged follow-up with information on stroke subclass was not available for all trials and the mean age of participants in this meta-analysis was 79.5 years, meaning extrapolating to different populations is not possible. In order for clinicians to be able to make clear future comparisons we would encourage future full reporting of the breakdown of stroke subtype.

## Conclusions

In this meta-analysis of randomised clinical trials, treatment of aortic stenosis with TAVI compared with SAVR was associated with no overall reduced risk in fatal or disabling stroke. However, subgroup analyses suggested a lower risk of fatal or disabling stroke with TAVI in situations which reflect contemporary practice. While the absolute differences may be small this still has important implications for choice of procedure for patients in the setting of widening indications for TAVI.

## Declaration of Competing Interest

None.

## Appendix Supplementary materials

Image, application 1
