# Supplementary material for: Stroke Severity in Transcatheter Aortic Valve Implantation Versus Surgical Aortic Valve Replacement: A Systematic Review and Meta-Analysis
Source: J Stroke Cerebrovasc Dis. 2021 Sep;30(9):None. doi: 10.1016/j.jstrokecerebrovasdis.2021.105927 (PMC8600126; doi:10.1016/j.jstrokecerebrovasdis.2021.105927)
Supplement: Supplementary file 1 [file mmc1.docx]

Supplementary appendix

Table of Contents

[Search strategy 2](#_Toc57226759)

[Figures 3](#_Toc57226760)

[eFigure 1 – Prisma Flow Diagram 3](#_Toc57226761)

[eFigure 2 – All stroke outcomes at between Year 1 and Year 2 4](#_Toc57226762)

[eFigure 3 – 30-day All Stroke: TAVI vs SAVR 5](#_Toc57226763)

[eFigure 4 – 1 year All Stroke: TAVI vs SAVR 6](#_Toc57226764)

[eFigure 5 – Risk of Bias 7](#_Toc57226765)

[eFigure 6 – Risk of Bias 8](#_Toc57226766)

## Search strategy

Cochrane CENTRAL: (October 31^st^ 2019)

(Aortic stenosis OR aorta stenosis OR Aortic Valve Stenoses OR aortic valve stenosis)

AND

(aortic valve implantation OR heart valve implantation OR TAVR OR TAVI OR transcatheter OR transfemoral OR transapical OR transaxillary OR SAVR OR heart valve replacement OR surgical aortic valve replacement OR surgical AVR OR SAVR)

In: Title, abstract, keywords

Limits: Publication Year from 2016, in Trials

Ovid Medline (October 31^st^ 2019) and Medline in-process (October 31^st^ 2019)

1. Aortic Stenosis.mp. or exp Aortic Valve Stenosis/

2. (aortic valve implantation or TAVR or transcatheter or transfemoral or transapical or transaxillary or SAVR or heart valve replacement or surgical aortic valve replacement or surgical AVR or SAVR or TAVI or aortic valve replacement or transvascular).af.

3. clinical trial.mp. or clinical trial.pt. or random:.mp. or tu.xs.

4. 1 and 2 and 3

5. limit 4 to yr="2016 -Current"

6. limit 5 to humans

Ovid EMBASE (October 31^st^ 2019)

1. aortic stenosis.mp. or exp aorta stenosis/

2. (aortic valve implantation or heart valve implantation or TAVR or TAVI or transcatheter or transfemoral or transapical or transaxillary or SAVR or heart valve replacement or surgical aortic valve replacement or surgical AVR or SAVR or aortic valve replacement or transvascular).af.

3. random:.tw. or placebo:.mp. or double-blind:.tw.

4. 1 and 2 and 3

5. limit 4 to yr="2016 -Current"

6. limit 5 to human

# Figures

## eFigure 1 ­– Prisma Flow Diagram

##

Full-text articles excluded, with reasons:

Not RCTs comparing TAVI with SAVR
(n = 29)

Studies included in quantitative synthesis (meta-analysis)
(n = 8 )

Studies included in qualitative synthesis
(n = 8)

Full-text articles assessed for eligibility
(n = 37 )

Records excluded
(n =172 )

Records screened
(n = 209 )

Records after duplicates removed
(n =209 )

Additional records identified through other sources
(n = 5)

Identification

Eligibility

Included

Screening

Records identified through database searching
(n = 227 )

## eFigure 2 ­– All stroke outcomes at between Year 1 and Year 2


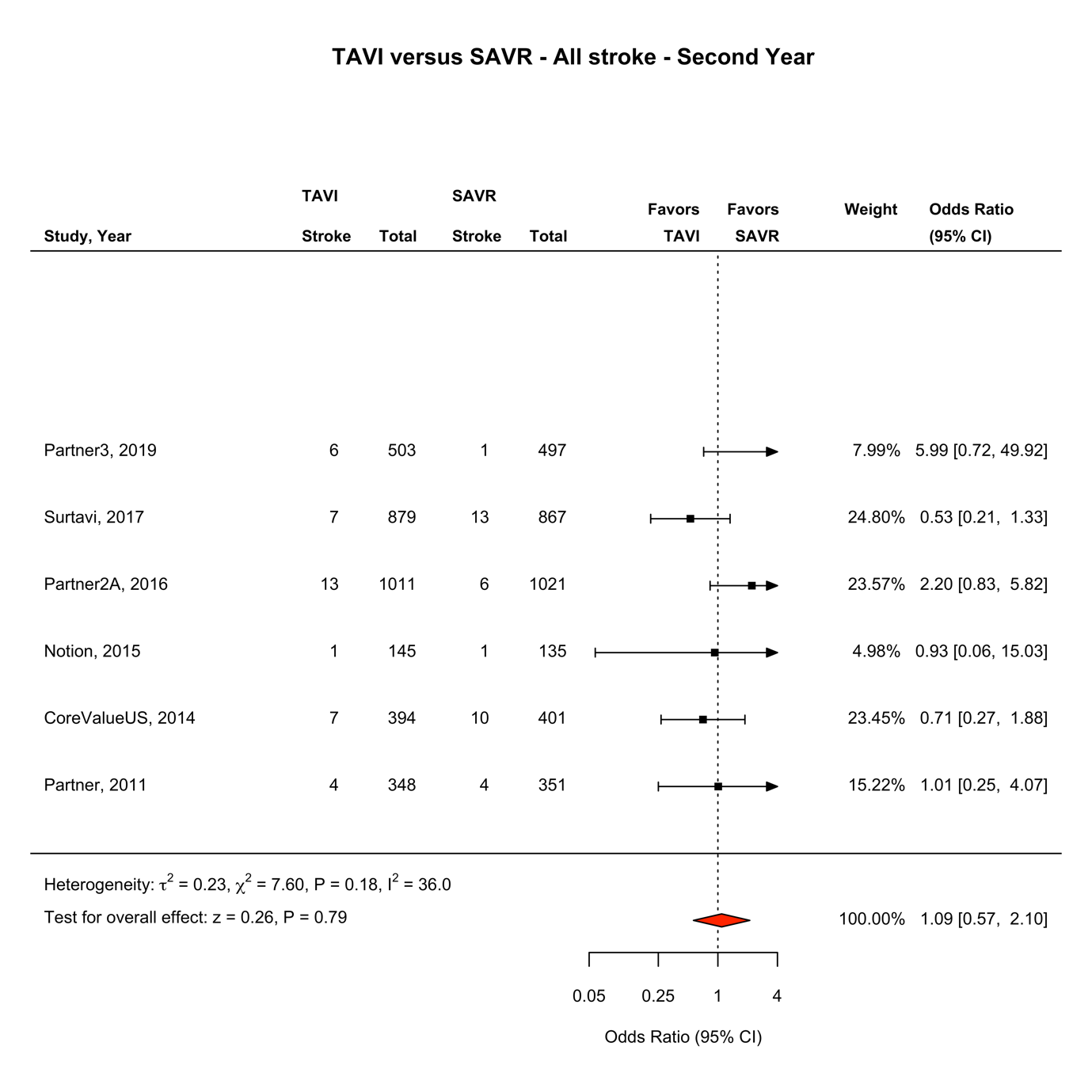


eFigure 2: All stroke outcomes at between year 1 and year 2. Forest Plot showing all stroke events by treatment group between year 1 and year 2. The square bars represent the mean values and 95% confidence interval of the effect sizes, while the size of the squares reflects the weight of the studies. The combined effect appears as a diamond and the vertical dashed line represents the line of no effect. Abbreviations: TAVI, transcatheter aortic valve implantation; SAVR, surgical aortic valve repair; CI, Confidence Interval

## eFigure 3 – 30-day All Stroke: TAVI vs SAVR


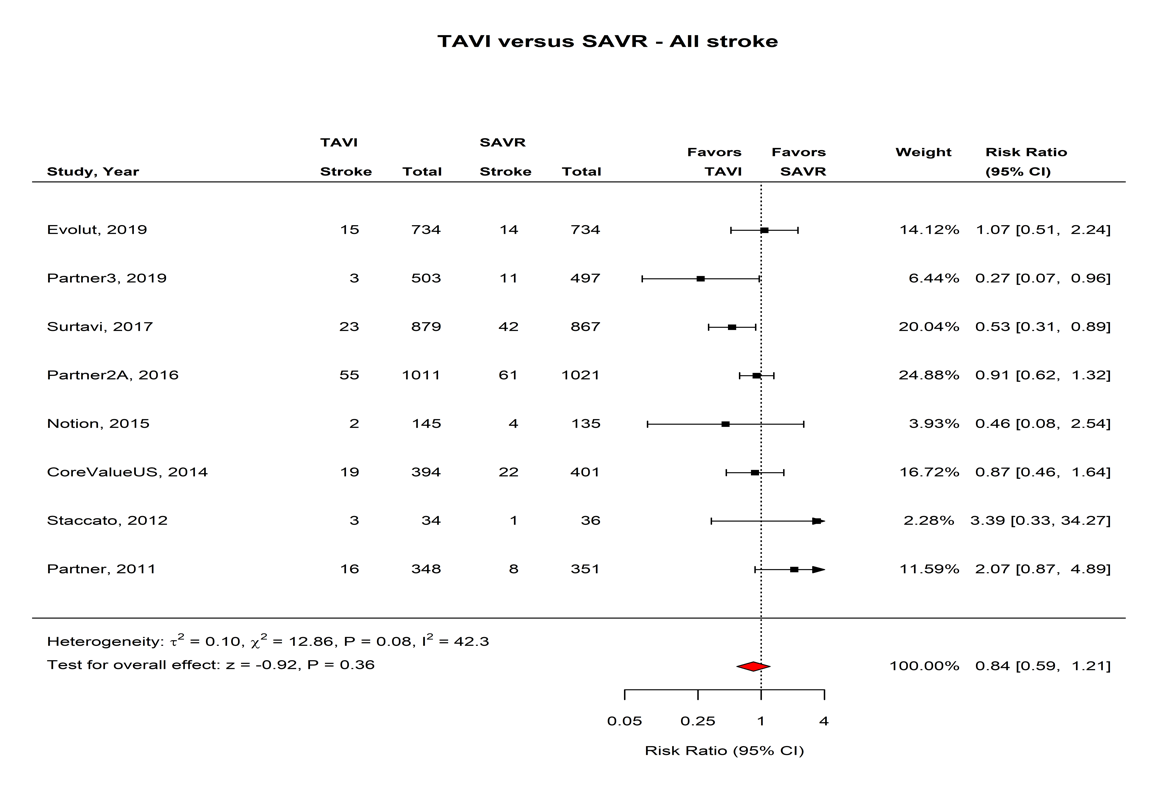


eFigure 3: 30 day all stroke events. Forest Plot showing all stroke events by treatment group at 30 days. The square bars represent the mean values and 95% confidence interval of the effect sizes, while the size of the squares reflects the weight of the studies. The combined effect appears as a diamond and the vertical dashed line represents the line of no effect. Abbreviations: TAVI, transcatheter aortic valve implantation; SAVR, surgical aortic valve repair; CI, Confidence Interval.

## eFigure 4 – 1 year All Stroke: TAVI vs SAVR


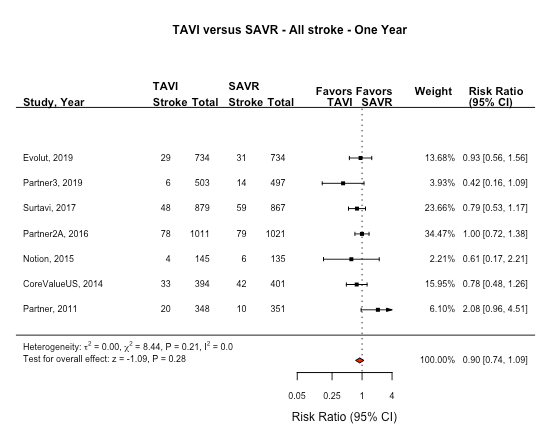


eFigure 4: One year all stroke events. Forest Plot showing all stroke events by treatment group at one year. The square bars represent the mean values and 95% confidence interval of the effect sizes, while the size of the squares reflects the weight of the studies. The combined effect appears as a diamond and the vertical dashed line represents the line of no effect. Abbreviations: TAVI, transcatheter aortic valve implantation; SAVR, surgical aortic valve repair; CI, Confidence Interval

## eFigure 5 – Risk of Bias


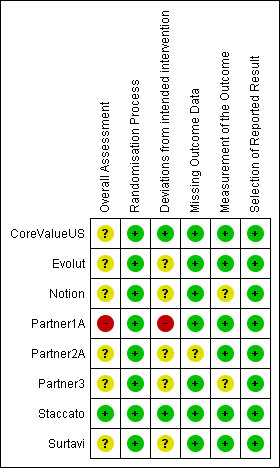


eFigure 5: Risk of bias summary. eFigure 5 was generated using the Cochrane Risk of Bias 2 tool.

https://methods.cochrane.org/bias/resources/rob-2-revised-cochrane-risk-bias-tool-randomized-trials

## eFigure 6 – Risk of Bias


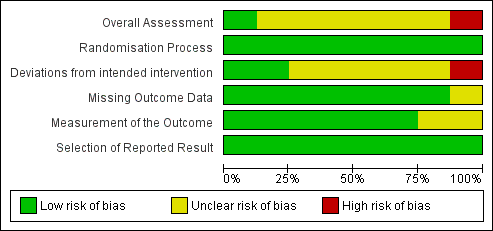


eFigure 6: Risk of bias crosstabulation. eFigure 6 was generated using the Cochrane Risk of Bias 2 tool.

https://methods.cochrane.org/bias/resources/rob-2-revised-cochrane-risk-bias-tool-randomized-trials
